# ABO blood group and risk of newly diagnosed nonalcoholic fatty liver disease: A case-control study in Han Chinese population

**DOI:** 10.1371/journal.pone.0225792

**Published:** 2019-12-04

**Authors:** Guo-Chao Zhong, Shan Liu, Yi-Lin Wu, Mei Xia, Jin-Xian Zhu, Fa-Bao Hao, Lun Wan

**Affiliations:** 1 Graduate School, Chongqing Medical University, Chongqing, China; 2 Department of Pediatrics, the People’s Hospital of Dazu District, Chongqing, China; 3 Department of Hepatobiliary Surgery, the Second Affiliated Hospital of Chongqing Medical University, Chongqing, China; 4 Pediatric Surgery Center, Qingdao Women and Children’s Hospital, Qingdao University, Qingdao, Shandong, China; 5 Department of Hepatobiliary Surgery, the People’s Hospital of Dazu District, Chongqing, China; Medizinische Fakultat der RWTH Aachen, GERMANY

## Abstract

**Background:**

ABO blood group has been associated with cardiovascular disease and cancer. However, whether ABO blood group is associated with nonalcoholic fatty liver disease (NAFLD) remains unknown. The present study aimed to clarify this issue.

**Methods:**

A hospital-based case-control study was performed in southwestern China. A total of 583 newly ultrasound-diagnosed NAFLD cases and 2068 controls were included. The adjusted odds ratios (ORs) and 95% confidence intervals (CIs) of developing NAFLD were calculated by multivariate logistic regression. A propensity score was developed for adjustment and matching.

**Results:**

The proportions of blood groups A, B, AB and O were 31%, 26%, 8% and 35%, respectively. Non-O blood groups were found to be significantly associated with an increased risk of NAFLD (the fully adjusted OR = 1.51, 95% CI: 1.19, 1.91); moreover, compared with blood group O, the fully adjusted ORs of developing NAFLD were 1.50 (95% CI: 1.13, 1.99) for blood group A, 1.59 (95% CI: 1.19, 2.14) for blood group B, and 1.37 (95% CI: 0.86, 2.18) for blood group AB. Similar results were obtained in both propensity-score-adjusted and propensity-score-matched analyses. No evidence of significant effect modification for the association of ABO blood group with the risk of NAFLD was found (all *P*_interaction_>0.05).

**Conclusions:**

Non-O blood groups are significantly associated with an increased risk of NAFLD. Our findings provide some epidemiological evidence for a possible role of ABO glycosyltransferase in the pathogenesis of NAFLD. However, these findings need to be validated by future studies.

## Introduction

Nonalcoholic fatty liver disease (NAFLD), a precursor of the metabolic syndrome, is characterized by excessive hepatic lipid accumulation in the absence of secondary causes of fatty liver [1]. NAFLD is now considered a major cause of chronic liver disease worldwide, with the global prevalence of up to 25.24% [2]. In addition to its potential to cause liver cirrhosis and hepatocellular carcinoma, NAFLD is a well-established risk factor for type 2 diabetes [3], chronic kidney disease [4], and cardiovascular disease (CVD) [5].

The ABO blood group system, the first genetic polymorphism discovered among humans, is divided into blood groups A, B, AB and O. In addition to red blood cells, various human cells and tissues also express ABO antigens, including vascular endothelium and platelets [6]. Beyond its relevance to immunohematology and transfusion medicine, ABO blood group has been associated with risks of CVDs, including stroke [blood group AB versus O: hazard ratio = 1.83, 95% confidence interval (CI): 1.01, 3.30] [7], venous thromboembolism (non-O blood groups versus O: incidence rate ratio = 1.80, 95% CI: 1.71, 1.88) [8], and coronary heart disease [blood group A versus O: odds ratio (OR) = 1.29, 95% CI: 1.12, 1.49; blood group B versus O: OR = 1.18, 95% CI: 1.02, 1.36; blood group AB versus O: OR = 1.27, 95% CI: 1.01, 1.60] [9]. In general, compared with subjects with blood group O, those with non-O blood groups have a higher risk of CVD. A possible mechanism underlying the above-mentioned association between ABO blood group and the risk of CVD may be attributable to increased levels of low-density lipoprotein cholesterol, inflammatory cytokines, and von Willebrand factor (vWF) and factor VIII in individuals with non-O blood groups comparted with those with blood group O [9, 10]. Interestingly, it was found that individuals with cardiovascular risk factors, including obesity, smoking, lack of exercise, hyperlipidemia, and type 2 diabetes [1, 11, 12], were also at an increased risk of NAFLD, indicating that CVD and NAFLD may share common etiologies. With these considerations in mind, a reasonable speculation is that non-O blood groups are possibly associated with an increased risk of NAFLD. Moreover, epidemiological studies have found that subjects with non-O blood groups have a higher level of total cholesterol [11] and a higher risk of type 2 diabetes [13] relative to those with blood group O, which also supports, at least in part, an association of ABO blood group with the risk of NAFLD, considering the roles of cholesterol and type 2 diabetes in the development of NAFLD [14, 15].

To the best of our knowledge, the association between ABO blood group and the risk of NAFLD has not been evaluated. Therefore, we conducted a case-control study in Han Chinese population to examine the hypothesis that non-O blood groups are associated with an increased risk of NAFLD.

## Methods

This study was approved by the Institutional Review Board of the Second Affiliated Hospital of Chongqing Medical University (approval number: RER2018-008), and was performed in accordance with the Declaration of Helsinki. Because the present study is a retrospective observational study using existing clinical data, the requirement for informed consent was waived for all patients. The results of current study were reported in accordance with the Strengthening the Reporting of Observational Studies in Epidemiology (STROBE) statement (S1 File).

### Study population

This is a hospital-based case-control study performed at a university-affiliated tertiary teaching hospital. An electronic database of inpatients (UniDMR Integrated Platform) was prospectively established from October 2014 onward to improve clinical practice and research. Between January 1, 2015 and December 31, 2016, there were a total of 123243 consecutive patients admitted to the hospital. Of them, 4611 and 133 inpatients had a discharge diagnosis of fatty liver and nonalcoholic steatohepatitis (NASH), respectively.

To reduce the potential impact of comorbidity on outcomes of interest, and to identify eligible cases [16] and controls, all patients with any of the following conditions were excluded: (a) malignancy or a history of malignancy, organ failure, acquired immune deficiency syndrome, pregnancy and lactation; (b) conditions in relation to hepatic steatosis (i.e., drug-induced liver disease or a history of using hepatotoxic medications, gallstone disease, total parenteral nutrition, Wilson’s disease, chronic hepatitis C); (c) excessive alcohol consumption (pure alcohol >140 g/week in men and >70 g/week in women) [16] or a history of alcohol drinking; (d) liver abscess or cyst, biliary tract disease, liver cirrhosis or hepatic encephalopathy; (e) missing data on ABO blood group testing or abdominal ultrasonography.

### Case and control selection

For case selection, we excluded patients with previously diagnosed fatty liver on admission to minimize the risk of Neyman bias. In addition, to reduce the risk of ascertainment bias, we asked experienced ultrasonographists to specifically review ultrasound films to validate the diagnosis of fatty liver. Thus, a total of 583 patients with newly ultrasound-diagnosed NAFLD (including 13 NASH cases) were eligible for inclusion. These patients were admitted to the hospital for various conditions, including abnormal alanine aminotransferase (ALT) and/or aspartate aminotransferase (AST) levels (16 patients).

**Fig 1:**
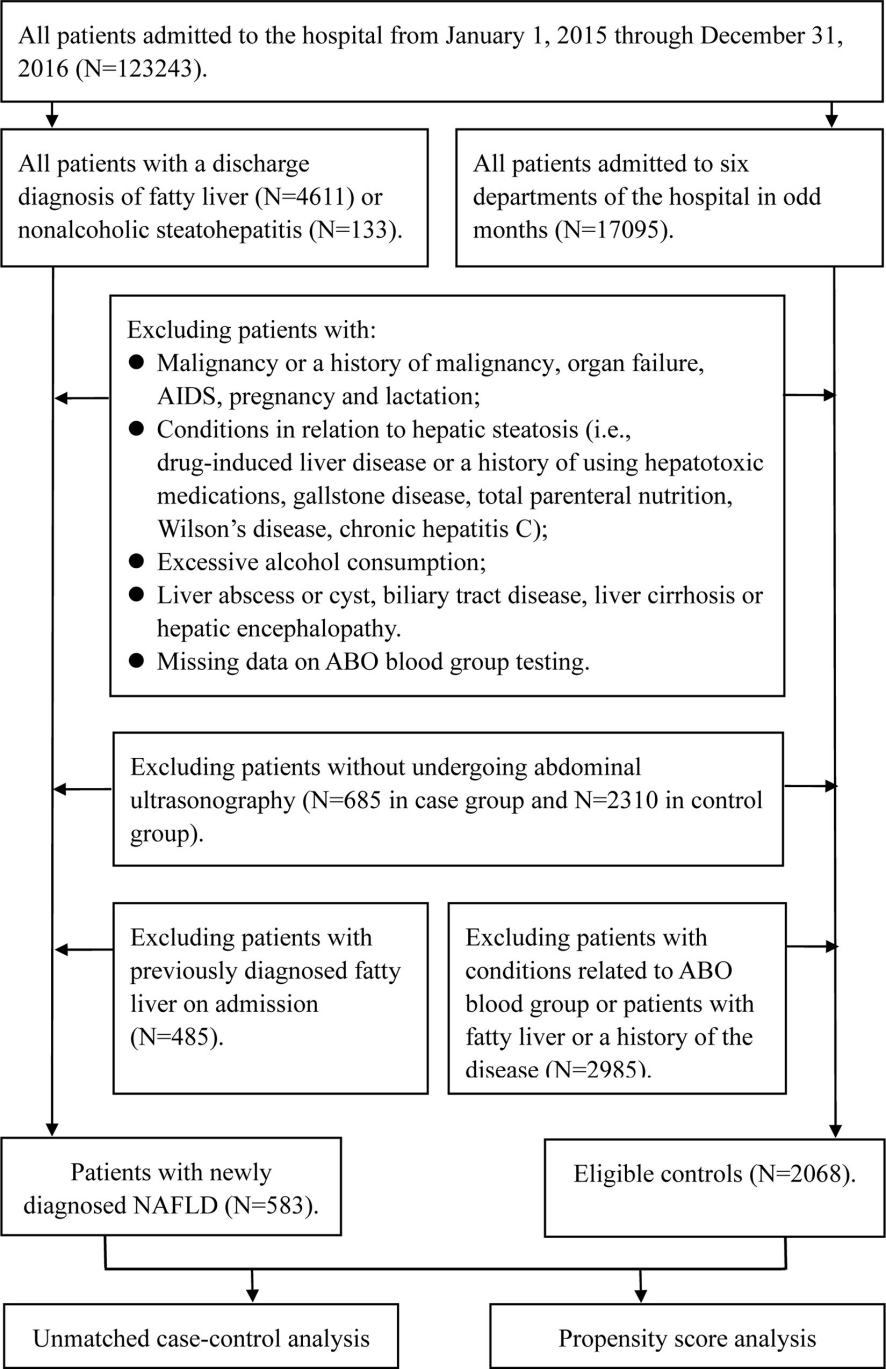
The study flowchart for identifying cases and controls. AIDS, acquired immune deficiency syndrome; NAFLD, nonalcoholic fatty liver disease.

Of 583 cases, 521 (89.4%) were from the Departments of Otolaryngology (31.2%), Gerontology (16.6%), Gastroenterology (14.0%), Cardiology (11.6%), Endocrinology (10.8%) and Nephrology (5.2%). Thus, for identifying eligible controls while minimizing workload and bias, we decided to choose controls among the patients admitted to these six departments in odd-numbered months during the same period as cases. After exclusion of patients admitted for conditions associated with ABO blood group and those with fatty liver or a history of the disease, we obtained a total of 2068 eligible controls. It is noteworthy that all eligible controls underwent abdominal ultrasonography scanning on admission, and corresponding ultrasound films were evaluated by experienced ultrasonographists to confirm the absence of fatty liver. These eligible controls were admitted to our hospital for the following reasons: otolaryngological diseases in 812, gastrointestinal diseases in 445, primary hypertension in 232, cardiac causes in 188, renal diseases in 161, thyroid disease in 85, immunological diseases 82, respiratory diseases in 32 and neurological diseases in 31. Fig 1 shows the process of patient selection.

### Study definitions

In the present study, fatty liver was diagnosed based on abdominal ultrasonography that patients underwent on admission. Because subjects with substantial alcohol consumption (or a history of drinking) and other conditions in relation to hepatic steatosis were excluded as mentioned above, the remaining fatty liver cases were considered NAFLD [16]. Any subject whose NAFLD activity score was more than four points was diagnosed as having NASH [16]. Controls were defined as those without fatty liver diagnosed by abdominal ultrasonography. Coronary heart disease and diabetes were diagnosed based on the diagnostic criteria of the World Health Organization [17, 18]. Hypertension was diagnosed according to Chinese guidelines for the management of hypertension [19].

### Data collection

Data on demographics, clinical features and laboratory tests were collected from the electronic database of the hospital (UniDMR Integrated Platform) by trained medical graduates who were blinded to the study protocol.

Demographics included age, sex, education level, and body mass index (BMI). Education level was divided into high (university or more), medium (junior or senior high school) or low (illiteracy or primary school). BMI was calculated as body weight (kg) divided by height squared (m^2^). Body weight and height were measured without thick clothing or shoes, or were collected by clinicians through face-to-face interview. As there were a total of 730 patients with missing data on height (152 in cases and 578 in controls), we sought to interview them via telephone to obtain these data, with an overall response rate of 54.5% (52.6% in cases and 55.0% in controls).

Clinical features included systolic and diastolic blood pressure, smoking status, alcohol consumption and medical histories. Blood pressure was measured in a sitting position after more than 10 minutes of rest by nurses using an electronic sphygmomanometer. Smoking status was categorized as never, past (stopped smoking ≥6 months ago) or current. Alcohol consumption was recorded as never, past (quit drinking ≥6 months ago) or current; both the average amount (pure alcohol, g/day) and the length of alcohol drinking were further collected for current drinkers. Medical histories included histories of coronary heart disease, hypertension, diabetes, infectious disease, and medication use. Data regarding smoking status, alcohol consumption and medical histories were collected by trained clinicians via face-to-face interviews.

Laboratory tests included fasting blood glucose (FBG), ALT, AST, alkaline phosphatase (ALP), γ-glutamyltransferase (γ-GTT), albumin, prothrombin time, total bilirubin, bile acid, triglycerides, total cholesterol, low-density lipoprotein cholesterol (LDL-C), high-density lipoprotein cholesterol (HDL-C), apolipoprotein E, and ABO phenotype. FIB-4 index was used to predict liver fibrosis of participants; it was calculated as age (years)×AST [U/L]/ (platelets [10^9^/L] × (ALT [U/L])^1/2^) [20]. Biochemical parameters described above were measured with automatic analyzers in clinical laboratories at the hospital. ABO blood typing was completed by the Department of Blood Transfusion using standard serologic methods for clinical purposes [21].

### Statistical analysis

Statistical analyses were conducted using R version 3.4.3 (The R Foundation for Statistical Computing, Vienna, Austria). The statistical significance level was set at *P*<0.05 under a two-tailed test. Since all continuous variables are not normally distributed as suggested by the Kolmogorov-Smirnov normality test (all *P*<0.01), they are expressed as medians (interquartile range), and categorical variables are expressed as counts (percentage). Between-group differences in demographic, clinical and laboratory characteristics were compared using the Wilcoxon rank-sum test for continuous variables and the χ^2^ test for categorical variables.

For minimizing potential bias and maximizing statistical power, we employed multiple imputation by chained equations to impute missing data under the assumption that the data are missing at random (the number of imputations = 5). The imputation model included following variables: age, sex, education level, diabetes, hypertension, coronary heart disease, smoking, albumin, ALT, AST, FIB-4 index, γ-GTT, total bilirubin, triglycerides, total cholesterol, LDL-C, HDL-C, ABO phenotype and NAFLD. Main analyses were repeated in patients with complete data for comparison. S1 Table presents the number with missing data and variable values before and after multiple imputation.

Multivariate logistic regression models were employed to estimate adjusted ORs and 95% CIs of developing NAFLD. Covariate selection for multivariate models was based on the clinical relevance and the change-in-estimate criterion [22]. Specifically, Model 1 adjusted for age (continuous) and sex (male, female). Model 2 further adjusted for education level (low, medium, high), BMI (continuous), diabetes (yes, no), hypertension (yes, no), smoking status (current, past, never), and triglycerides (continuous). Model 3 adjusted for all variables selected by the change-in-estimate criterion. Model 4 adjusted for all variables mentioned above as well as FIB-4 index (continuous).

To identify potential effect modifiers of the association between ABO blood group and the risk of NAFLD, we performed a series of a priori subgroup analyses stratified by age (≥60 vs <60 years), sex (male vs female), body mass index (BMI) (≥25 vs <25 kg/m^2^), current smoking (yes vs no), diabetes (yes vs no), hypertension (yes vs no), ALT (>40 vs ≤40 U/L), fasting blood glucose (FBG) (≥7.0 vs <7.0 mmol/L), and triglycerides (≥1.7 vs <1.7 mmol/L). Note that subgroup analyses were based on the most fully adjusted risk estimates for the association of non-O blood groups with the risk of NAFLD. We tested the significance of interaction between ABO blood group (non-O blood groups versus O) and the above-mentioned stratification factors using a likelihood ratio test that compares models with and without interaction terms.

We further performed propensity-score-adjusted and propensity-score-matched analyses to examine the association between ABO blood group and the risk of NAFLD. The propensity score was calculated for each patient of case and control groups using a multivariate logistic regression model, which contained 18 covariates associated with the development of NAFLD, namely, age, sex, BMI, diabetes, hypertension, smoking, prothrombin time, FBG, ALT, albumin, AST, FIB-4 index, γ-GTT, total cholesterol, triglycerides, LDL-C, HDL-C and apolipoprotein E. The *c*-statistic was employed to quantitatively evaluate the discrimination ability of the propensity score model. In the present study, the *c*-statistic for the propensity model was 0.86. A 1:1 greedy nearest-neighbor matching approach without replacement was employed in our propensity-score-matched analysis, with a caliper width equal to 0.2 of the standard deviation of the logit of the propensity score (caliper = 0.05) [23].

## Results

### Patient characteristics

The demographic, clinical and laboratory characteristics of the study population after multiple imputation are shown in Table 1. No significant differences were found between cases and controls in terms of age, education level, prevalence of coronary heart disease, platelet count, FIB-4 index, and total bilirubin level (all *P*>0.05). However, compared with controls, NAFLD cases were more likely to be male, obese and smokers, and to have diabetes and hypertension (all *P*<0.05). In addition, NAFLD cases had significantly higher concentrations of FBG, ALT, AST, ALP, γ-GTT, albumin, bile acid, triglycerides, total cholesterol, LDL-C and apolipoprotein E than did controls (all *P*<0.05). In our study population, the proportions of blood groups O, A, B, and AB were 35%, 31%, 26%, and 8%, respectively. Interestingly, when grouping the study population by the blood group, we found that there was no significant difference in the above-mentioned characteristics except the prevalence of diabetes and the level of ALP among four groups (S2 Table).

**Table 1:** Demographic, clinical and laboratory characteristics of patients after multiple imputation.

Characteristics ^a^	Cases (N = 583)	Controls (N = 2068)	*P*
Demographics			
Age (years)	57 (47–65)	58 (45–67)	0.527
Male	255 (43.7)	777 (37.6)	0.007
Education			
Low	110 (18.9)	439 (21.2)	0.424
Medium	323 (55.4)	1096 (53.0)	
High	150 (25.7)	533 (25.8)	
Body mass index (kg/m^2^)	26.0 (24.1–28.0)	22.5 (20.4–24.5)	<0.001
Clinical features			
SBP (mm Hg)	131 (121–144)	126 (117–139)	<0.001
DBP (mm Hg)	80 (74–90)	78 (70–85)	<0.001
Smoking status			
Never	450 (77.2)	1720 (83.2)	0.001
Past	21 (3.6)	74 (3.6)	
Current	112 (19.2)	274 (13.3)	
Coronary heart disease	136 (23.3)	410 (19.8)	0.065
Hypertension	273 (46.8)	593 (28.7)	<0.001
Diabetes	180 (30.9)	314 (15.2)	<0.001
Laboratory tests			
Platelet count (10^9^/L)	194 (157–233)	191 (156–228)	0.438
FBG (mmol/L)	5.75 (5.02–7.25)	5.00 (4.52–5.70)	<0.001
ALT (U/L)	26 (18–43)	16 (12–24)	<0.001
AST (U/L)	23 (18–30)	20 (17–25)	<0.001
FIB-4 index ^b^	1.33 (0.95–1.84)	1.45 (0.98–2.05)	0.527
ALP (U/L)	80 (66–93)	74 (61–89)	<0.001
γ-GTT (U/L)	32 (21–52)	19 (14–27)	<0.001
Albumin (g/L)	43.1 (40.9–45.2)	42.5 (40.1–44.6)	<0.001
Prothrombin time (s)	12.8 (12.4–13.3)	13.1 (12.6–13.6)	<0.001
Total bilirubin (umol/L)	10.7 (8.4–13.6)	10.5 (8.1–13.4)	0.211
Bile acid (umol/L)	3.7 (2.2–6.3)	3.4 (2.1–5.7)	0.042
Triglycerides (mmol/L)	1.79 (1.30–2.71)	1.14 (0.84–1.61)	<0.001
Total cholesterol (mmol/L)	4.78 (4.16–5.55)	4.52 (3.91–5.22)	<0.001
LDL-C (mmol/L)	2.79 (2.27–3.42)	2.57 (2.05–3.11)	<0.001
HDL-C (mmol/L)	1.07 (0.91–1.25)	1.21 (1.01–1.47)	<0.001
Apolipoprotein E (mg/L)	40.0 (33.9–47.0)	35.9 (31.1–42.2)	<0.001

SBP, systolic blood pressure; DBP, diastolic blood pressure; FBG, fasting blood glucose; ALT, alanine aminotransferase; AST, aspartate aminotransferase; ALP, alkaline phosphatase; γ-GTT, γ-glutamyltransferase; LDL-C, low-density lipoprotein cholesterol; HDL-C, high-density lipoprotein cholesterol.

^a^ Data are median (interquartile range) or N (%) as indicated

^b^ FIB-4 index is a simple noninvasive index to predict liver fibrosis and is calculated as age (years)×AST [U/L]/ (platelets [10^9^/L] × (ALT [U/L])^1/2^).

### Unmatched case-control analysis

In univariate analysis, we found that patients with non-O blood groups had a significantly higher risk of NAFLD than those with blood group O (OR = 1.22, 95% CI: 1.00, 1.48) (Table 2). In the fully adjusted model (model 4), the adjusted OR of developing NAFLD among patients with non-O blood groups was 1.51 (95% CI: 1.19, 1.91). Similar results were obtained for blood groups A and B in univariate and multivariate analyses. In the fully adjusted model, both blood groups A (OR = 1.50, 95% CI: 1.13, 1.99) and B (OR = 1.59, 95% CI: 1.19, 2.14) were found to be individually associated with an increased risk of NAFLD. We did not find a significant association between blood group AB and the risk of NAFLD in either the univariate or multivariate analysis. When repeating the aforementioned analyses among patients with complete data, we obtained similar results (S3 Table).

**Table 2:** Results of univariate and multivariate analyses on ABO blood group and risk of nonalcoholic fatty liver disease.

Blood group	Cases ^a^	Controls ^a^	Odds ratio (95% confidence interval)				
			Unadjusted	Model 1	Model 2	Model 3	Model 4
O	187 (32.1)	756 (36.6)	1.00 (reference)	1.00 (reference)	1.00 (reference)	1.00 (reference)	1.00 (reference)
Non-O	396 (67.9)	1312 (63.4)	1.22 (1.00–1.48)	1.26 (1.03–1.53)	1.52 (1.21–1.93)	1.50 (1.18, 1.89)	1.51 (1.19, 1.91)
A	194 (33.3)	630 (30.4)	1.24 (0.99–1.56)	1.29 (1.03–1.62)	1.53 (1.16–2.00)	1.50 (1.13, 1.99)	1.50 (1.13, 1.99)
B	162 (27.8)	523 (25.3)	1.25 (0.99–1.59)	1.27 (1.00–1.61)	1.57 (1.19–2.09)	1.59 (1.19, 2.13)	1.59 (1.19, 2.14)
AB	40 (6.8)	159 (7.7)	1.02 (0.69–1.49)	1.07 (0.73–1.58)	1.34 (0.85–2.12)	1.37 (0.86, 2.17)	1.37 (0.86, 2.18)

Model 1 adjusted for age (continuous) and sex (male, female). Model 2 further adjusted for education level (low, medium, high), BMI (continuous), diabetes (yes, no), hypertension (yes, no), smoking status (current, past, never), and triglycerides (continuous). Model 3 adjusted for all variables selected by the change-in-estimate criterion [for non-O blood groups and risk of nonalcoholic fatty liver disease: BMI (continuous), diabetes (yes, no), smoking status (current, past, never), FBG (continuous), ALT (continuous), γ-GTT (continuous), triglycerides (continuous), and HDL-C (continuous); for blood groups A, B and AB and risk of nonalcoholic fatty liver disease: sex (male, female), education level (low, medium, high), BMI (continuous), diabetes (yes, no), hypertension (yes, no), coronary heart disease (yes, no), smoking status (current, past, never), prothrombin time (continuous), FBG (continuous), albumin (continuous), ALT (continuous), AST (continuous), ALP (continuous), γ-GTT (continuous), bile acid (continuous), triglycerides (continuous), LDL-C (continuous), HDL-C (continuous) and apolipoprotein E (continuous)]. Model 4 adjusted for all variables mentioned above as well as FIB-4 index (continuous).

^a^ Data are expressed as N (%).

**Fig 2:**
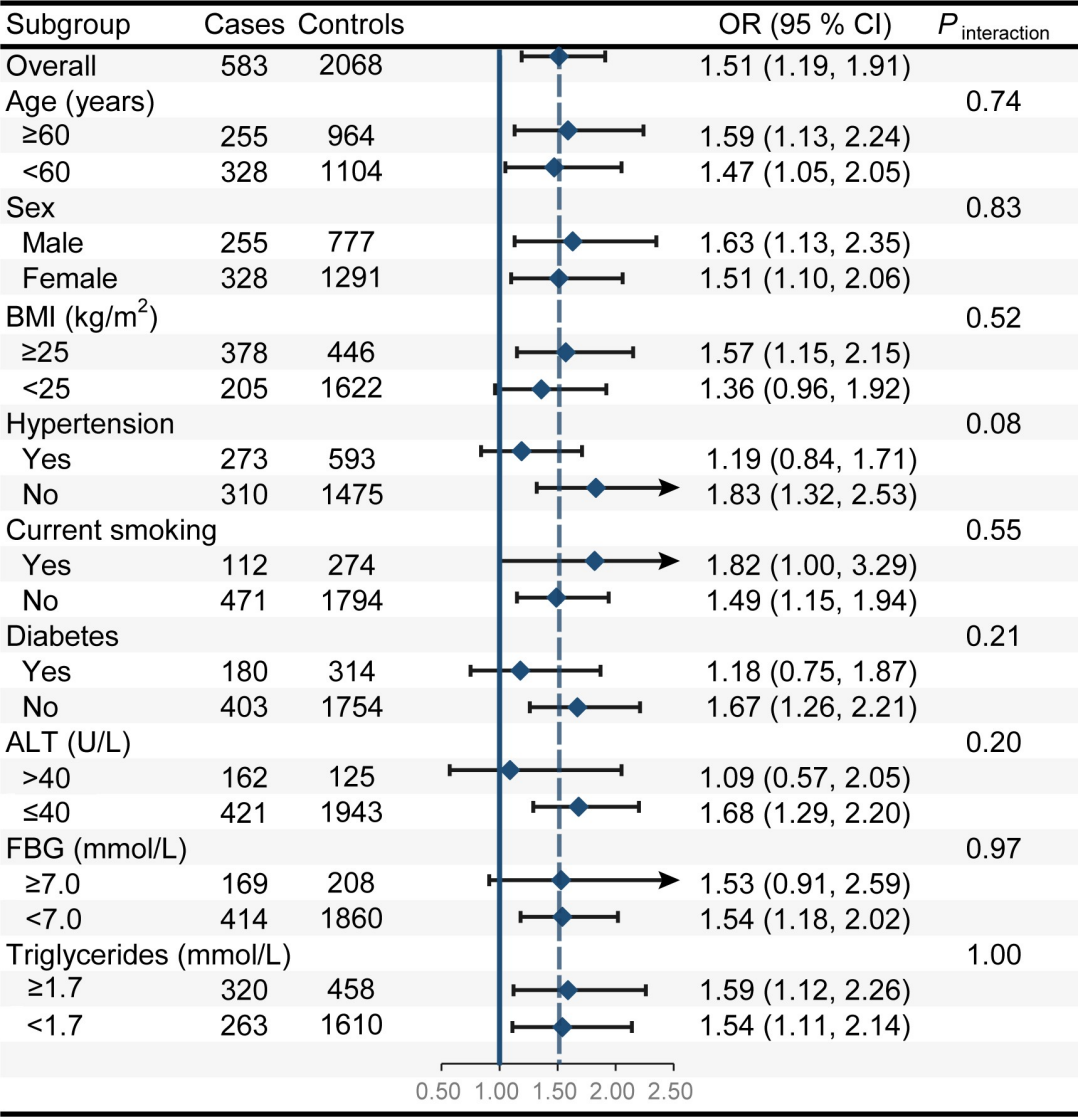
The results of subgroup analyses on the association of non-O blood groups with the risk of nonalcoholic fatty liver disease. These subgroup analyses were based on the most fully adjusted risk estimates (i.e., data from model 4). *P*_*i*nteraction_ was calculated from a likelihood ratio test. In each case, the model was not adjusted for the stratification factor. OR, odds ratio; CI, confidence interval; BMI, body mass index; ALT, alanine aminotransferase; FBG, fasting blood glucose.

We did not find evidence of significant effect modification by age, sex, BMI, current smoking, diabetes, hypertension, ALT, FBG, or triglycerides for the association of non-O blood groups with the risk of NAFLD (all *P*_interaction_>0.05) (Fig 2). Similarly, we did not find evidence of significant effect modification by the aforementioned stratification factors for the associations of blood groups A, B and AB with the risk of NAFLD (all *P*_interaction_>0.05) (S4 Table).

### Propensity score analysis

For obtaining effect sizes of the whole study population and improving the statistical power of our analysis, we first performed a propensity-score-adjusted analysis on the association of ABO blood group with the risk of NAFLD (Table 3). Our propensity-score-adjusted analysis showed that individuals with non-O blood groups had a significantly higher risk of NAFLD than those with blood group O (OR = 1.50, 95% CI: 1.18, 1.91). Of the different non-O blood groups, both blood groups A (OR = 1.47, 95% CI: 1.11, 1.93) and B (OR = 1.59, 95% CI: 1.18, 2.12) were significantly associated with an increased risk of NAFLD, while there was no significant association between blood group AB and the risk of NAFLD (OR = 1.36, 95% CI: 0.86, 2.15).

**Table 3:** Results of propensity score analyses on ABO blood group and risk of nonalcoholic fatty liver disease.

Blood group	Propensity-score-adjusted ^a^			Propensity-score-matched		
	Cases ^b^	Controls ^b^	OR (95% CI)	Cases ^b^	Controls ^b^	OR (95% CI)
**O**	187 (32.1)	756 (36.6)	1.00 (reference)	144 (30.8)	201 (42.9)	1.00 (reference)
**Non-O**	396 (67.9)	1312 (63.4)	1.50 (1.18, 1.91)	324 (69.2)	267 (57.1)	1.69 (1.30, 2.22)
**A**	194 (33.3)	630 (30.4)	1.47 (1.11, 1.93)	150 (32.1)	120 (25.6)	1.74 (1.26, 2.41)
**B**	162 (27.8)	523 (25.3)	1.59 (1.18, 2.12)	140 (29.9)	112 (23.9)	1.74 (1.26, 2.42)
**AB**	40 (6.8)	159 (7.7)	1.36 (0.86, 2.15)	34 (7.3)	35 (7.5)	1.36 (0.81, 2.28)

OR, odds ratio; CI, confidence interval.

^a^ Propensity score was adjusted as a continuous variable in the regression model.

^b^ Data are expressed as N (%)

To reduce the confounding effect derived from differences in demographic, clinical and laboratory characteristics between cases and controls, we next performed a propensity-score-matched analysis (Table 3). On the basis of data after multiple imputation, we obtained a total of 429 matched pairs consisting of one NAFLD case and one control. After matching, there was no significant difference in patient characteristics between cases and controls (all *P* >0.05) (S5 Table). Within the matched cohort, the aforementioned associations of non-O groups as well as blood groups A, B and AB with the risk of NAFLD remained. We repeated the above-mentioned propensity score analyses among individuals with complete data, and obtained similar results (S6 Table).

## Discussion

To the best of our knowledge, this is the first study to report an association of ABO blood group with the risk of NAFLD. Our unmatched case-control analysis showed that non-O blood groups were significantly associated with an increased risk of NAFLD, even after accounting for the relevant confounders, which was consistent with the results of both propensity-score-adjusted and propensity-score-matched analyses. Moreover, compared with individuals with blood group O, those with blood group A or B had a significantly higher risk of NAFLD, whereas those with blood group AB did not. Our subgroup analyses did not identify any effect modifiers of the association between ABO blood group and the risk of NAFLD.

According to amounts of antigens on the surface of red blood cell, blood group A is divided into two principle subtypes, namely A_1_ and A_2_, with A_1_ and A_2_ accounting for approximately 80% and 20% of blood group A, respectively. The serologic difference between subtypes A_1_ and A_2_ is that A_1_ but not A_2_ red blood cell can agglutinate with anti-A_1_ lectin [6]. In addition to the difference in serology, some epidemiological studies have revealed significant differences in biochemical parameters and risks of developing CVD and cancer between two subtypes. For example, individuals with subtype A_1_ were found to have higher plasma levels of total cholesterol, LDL-C, vWF and factor VIII [10, 24], and higher risks of venous thromboembolism [8] and pancreatic cancer [25] than those with subtype A_2_. These differences possibly result from a 30- to 50-fold lower catalytic activity of A_2_ glycosyltransferase than A_1_ glycosyltransferase [26]. Therefore, it is important and necessary to distinguish between subtypes A_1_ and A_2_ in observational and genetic studies. However, the present study did not check for the distinction between these subtypes, owing to the lack of ABO subtype data from the study population. Consequently, the impact of aforementioned differences between subtypes A_1_ and A_2_ on the risk of NAFLD remains unclear, and needs to be addressed in future studies.

The Rh blood group system plays an important role in transfusion medicine, and is composed of ≥45 independent antigens. The frequency of Rh blood group varies across different populations. A nationwide cross-sectional study in the Chinese population found that the percentage of Rh(D)-negative subjects was only 1.02% [27], whereas a large-scale study of American donors found that this percentage went up to 14.6% [28]. In the present study, considering a relatively low percentage of Rh(D)-negative subjects in the Chinese population, which directly results in insufficient statistical power for the association between Rh blood group and the risk of NAFLD, we did not evaluate the combined effect of ABO and Rh blood groups on the risk of NAFLD. Nevertheless, several studies with large sample size on Rh blood group system and health outcome consistently revealed a null association [13, 29–31], indirectly indicating that the direction and magnitude of the association between ABO blood group and NAFLD risk observed in our study may not be altered substantially after considering Rh blood group system.

The mechanisms of action underlying the association of ABO blood group with the risk of NAFLD remain unclear. Some studies have revealed an association of hepatic cholesterol metabolism and accumulation with liver injury and hepatocyte death in NAFLD [32–34], which suggests a critical role of cholesterol in the pathogenesis of the disease [15]; a prospective cohort study further showed that higher total cholesterol levels were associated with a higher risk of NAFLD [35]. It was found that individuals with non-O blood groups had a significantly higher level of total cholesterol relative to those with blood group O [9]. Altogether, we think that the increased risk of NAFLD associated with non-O blood groups can be only partly explained by their impacts on cholesterol levels. In fact, a mediation analysis showed that approximately 10% of the effect of non-O blood groups on the risk of coronary heart disease was mediated by increased LDL-C levels [9], which supports our aforementioned thought to some extent. An alternative mechanism for the observed association between ABO blood group and NAFLD risk possibly involves the fact that vWF levels are about 25% higher in subjects with non-O blood groups than those with blood group O [36]. This difference in vWF levels between two groups may be attributable to a functional effect of ABO locus itself [37] as well as the presence of ABO antigenic structures in asparagine-linked oligosaccharide chains of vWF [38]. Of note, patients with fatty liver have been found to have a higher level of vWF than those without [39]; an increasing level of vWF has been found to be significantly associated with metabolic features of NAFLD patients [40]. In addition, changes in the levels of inflammatory cytokines associated with ABO blood group may provide a further mechanism. A genome-wide association study identified that single-nucleotide polymorphisms at the ABO locus were associated with plasma levels of tumor necrosis factor-α [41], which was found to be a predictor for the development of NAFLD [42].

Our study has several limitations. First, owing to unrecognized or unmeasured confounders (e.g., vWF), our results may be affected by residual confounding. Second, our study is a single-center hospital-based study, which raises a concern regarding the representativeness of our study population. Therefore, the possibility of selection bias cannot be ruled out. In addition, abdominal ultrasonography is not routinely performed for inpatients in our hospital, which also raises the possibility of selection bias. Nevertheless, in our study, both NAFLD cases and controls were selected from the same hospital, and controls were also roughly matched to cases for the department, which reduce the risk of selection bias. Third, considering the different ABO phenotype distribution and genetic background across populations, our findings derived from Chinese patients might not be completely generalizable to other populations. Finally, in our study, the NAFLD diagnosis was based on abdominal ultrasonography rather than liver biopsy, leading to potential misclassification between cases and controls. Moreover, abdominal ultrasonography for the diagnosis of NAFLD presents a high inter-observer variability and has poor performance in staging of the disease. However, because of its invasiveness, liver biopsy cannot be widely used in mass screening. In addition, abdominal ultrasonography is a commonly used method to detect fatty liver in clinical practice and research, with high accuracy and reliability [43]; notably, in our study, we asked experienced ultrasonographists to specifically review ultrasound films to validate the diagnosis of fatty liver, indicating that the inter-observer variability might be low.

Generally, one would expect that most NAFLD patients are from the Department of Gastroenterology. Indeed, among 4611 patients with fatty liver admitted to our hospital, more than 50% were from the Department of Gastroenterology. However, of patients included in our study, nearly one-third of NAFLD patients were hospitalized in the Department of Otolaryngology, whereas only 14% of them were hospitalized in the Department of Gastroenterology. The main reason behind this phenomenon is that a large proportion of patients with fatty liver admitted to the Department of Gastroenterology had conditions in relation to hepatic steatosis or hepatobiliary diseases and were therefore excluded according to the predefined exclusion criteria. Of note, in the present study, most eligible patients had otolaryngological diseases, mainly including polyp of vocal cord, nasal polyp, hypertrophy of tonsil, chronic sinusitis, and acute epiglottitis, and received symptomatic treatment or surgery during hospitalization. The inclusion of a large number of patients with otolaryngological diseases raised a concern that selection bias might distort our results. Nevertheless, the frequency of ABO blood group observed in controls of our study was similar to that in the Chinese general population [27], which alleviates this concern to some extent. Based on a thorough literature review, we had verified that the aforementioned otolaryngological diseases were not known to be associated with ABO blood group, which further alleviates the concern of selection bias [44]. In addition, clinical managements during hospitalization could affect the amount of liver fat. Nevertheless, in our study, case and control selection was based on abdominal ultrasonography scanning on admission.

In conclusion, our results indicate that non-O blood groups are significantly associated with an increased risk of NAFLD in Han Chinese population. These results provide some epidemiological evidence for a possible role of ABO glycosyltransferase in the pathogenesis of NAFLD. Nevertheless, our results need to be confirmed by future studies with large sample size and good design. If confirmed, ABO blood group can potentially be added to existing prediction scores for NAFLD to improve their performance. In addition, the exact biological mechanisms underlying the association between ABO blood group and NAFLD risk remain to be elucidated.

## Supporting information

S1 File The STROBE checklist. (DOCX)

S1 Table Distribution of variables with missing data before and after multiple imputation. (DOC)

S2 Table Demographic, clinical and laboratory characteristics of patients among different blood groups after multiple imputation. (DOC)

S3 Table Results of univariate and multivariate analyses on ABO blood group and NAFLD risk in patients with complete data. (DOC)

S4 Table The results of subgroup analyses on the associations of blood groups A, B and AB with the risk of NAFLD.
(DOC)

S5 Table Demographic, clinical and laboratory characteristics of patients after multiple imputation and propensity score matching. (DOC)

S6 Table Results of propensity score analyses on ABO blood group and risk of nonalcoholic fatty liver disease in patients with complete data. (DOC)

S1 Dataset Original data associated with the present study. (XLSX)
