# Supplementary material for: ABO blood group and risk of newly diagnosed nonalcoholic fatty liver disease: A case-control study in Han Chinese population
Source: PLoS One. 2019 Dec 4;14(12):e0225792. doi: 10.1371/journal.pone.0225792 (PMC6892526; doi:10.1371/journal.pone.0225792)
Supplement: S1 Table — (DOC) [file pone.0225792.s002.doc]

| **S1 Table.** Distribution of variables with missing data before and after multiple imputation | | | |
| --- | --- | --- | --- |
| Variable ^a^ | Number (%) with missing data | Before multiple imputation | After multiple imputation |
| Body mass index (kg/m^2^) | 332 (12.52) | 23.2 (20.9–25.5) | 23.1 (20.9–25.5) |
| Prothrombin time (s) | 26 (0.98) | 13.0 (12.5–13.5) | 13.0 (12.5–13.5) |
| Fasting plasma glucose (mmol/L) | 31 (1.17) | 5.11 (4.59–6.00) | 5.12 (4.59–6.00) |
| Alkaline phosphatase (U/L) | 2 (0.08) | 75 (62–90) | 75 (62–90) |
| Bile acid (umol/L) | 19 (0.72) | 3.5 (2.1–5.8) | 3.5 (2.1–5.8) |
| Apolipoprotein E (g/L) | 18 (0.68) | 36.6 (31.7–43.2) | 36.6 (31.7–43.2) |

^a^ Data are median (interquartile range).
