# Supplementary material for: ABO blood group and risk of newly diagnosed nonalcoholic fatty liver disease: A case-control study in Han Chinese population
Source: PLoS One. 2019 Dec 4;14(12):e0225792. doi: 10.1371/journal.pone.0225792 (PMC6892526; doi:10.1371/journal.pone.0225792)
Supplement: S2 Table — (DOC) [file pone.0225792.s003.doc]

| **S2 Table.** Demographic, clinical and laboratory characteristics of patients among different blood groups after multiple imputation | | | | | |
| --- | --- | --- | --- | --- | --- |
| Characteristics ^a^ | Blood group | | | | *P* |
|  | O (N=943) | A (N=824) | B (N=685) | AB (N=199) |  |
| Demographics | | | | | |
| Age (years) | 59 (46–67) | 57 (45–67) | 57 (45–66) | 58(47–68) | 0.321 |
| Male | 377 (40.0) | 316 (38.3) | 261 (38.1) | 78 (39.2) | 0.860 |
| Education | | | | | |
| Low | 196 (20.8) | 171 (20.8) | 153 (22.3) | 29 (14.6) | 0.172 |
| Medium | 511 (54.2) | 423 (51.3) | 369 (53.9) | 116 (58.3) |  |
| High | 236 (25.0) | 230 (27.9) | 163 (23.8) | 54 (27.1) |  |
| Body mass index (kg/m^2^) | 23.2 (20.9–25.6) | 23.3 (20.8–25.5) | 23.0(21.0–25.4) | 22.8 (20.8–25.0) | 0.449 |
| Clinical features | | | | | |
| SBP (mm Hg) | 128(120–140) | 128 (117–140) | 127 (118–138) | 127 (118–138) | 0.681 |
| DBP (mm Hg) | 79 (71–86) | 78 (71–85) | 78 (71–85) | 79 (71–85) | 0.697 |
| Smoking status | | | | | |
| Never | 749 (79.4) | 692 (84.0) | 569 (83.1) | 160 (80.4) | 0.078 |
| Past | 33 (3.5) | 34 (4.1) | 20 (2.9) | 8 (4.0) |  |
| Current | 161 (17.1) | 98 (11.9) | 96 (14.0) | 31 (15.6) |  |
| Coronary heart disease | 204 (21.6) | 169 (20.5) | 127 (18.5) | 46 (23.1) | 0.367 |
| Hypertension | 315 (33.4) | 276 (33.5) | 211 (30.8) | 64 (32.2) | 0.660 |
| Diabetes | 152 (16.1) | 168 (20.4) | 128 (18.7) | 46 (23.1) | 0.041 |
| Laboratory tests | | | | | |
| Platelet count (10^9^/L) | 194 (157-231) | 190 (156-228) | 191 (155-228) | 198 (156-234) | 0.291 |
| FBG (mmol/L) | 5.10 (4.59–5.94) | 5.12 (4.62–6.06) | 5.11 (4.54–6.01) | 5.21 (4.65–5.86) | 0.506 |
| ALT (U/L) | 18 (13–28) | 17 (12–26) | 18 (13–26) | 18 (14–28) | 0.160 |
| AST (U/L) | 21 (18–26) | 20 (17–25) | 21(17–25) | 21(17–25) | 0.328 |
| FIB-4 index ^b^ | 1.43 (1.01-2.06) | 1.42 (0.96-1.95) | 1.39 (0.95-2.00) | 1.41 (0.92-2.03) | 0.512 |
| ALP (U/L) | 79 (66–94) | 68 (56–83) | 78 (66–93) | 72 (59–87) | <0.001 |
| γ–GTT (U/L) | 21 (15–34) | 21 (15–33) | 20 (15–31) | 22 (15–36) | 0.734 |
| Albumin (g/L) | 42.6 (40.5–44.5) | 42.3 (40.0–44.6) | 43.0 (40.7–45.1) | 42.4 (39.9–44.9) | 0.068 |
| Prothrombin time (s) | 13.0 (12.5–13.5) | 13.1(12.6–13.5) | 12.9 (12.5–13.5) | 13.0 (12.5–13.6) | 0.738 |
| Total bilirubin (umol/L) | 10.5 (8.2–13.3) | 10.6 (8.1–13.4) | 10.5 (8.3–13.5) | 10.5 (7.9–13.6) | 0.953 |
| Bile acid (umol/L) | 3.4 (2.1–5.7) | 3.5 (2.2–6.0) | 3.4 (2.0–5.7) | 4.1 (2.3–6.2) | 0.156 |
| Triglycerides (mmol/L) | 1.31 (0.92–1.92) | 1.20 (0.87–1.79) | 1.24 (0.92–1.78) | 1.22 (0.89–1.72) | 0.377 |
| Total cholesterol (mmol/L) | 4.55 (3.99–5.22) | 4.60 (3.94–5.36) | 4.64 (4.04–5.33) | 4.46 (3.94–5.24) | 0.876 |
| LDL–C (mmol/L) | 2.60 (2.08–3.16) | 2.60 (2.04–3.18) | 2.70 (2.11–3.18) | 2.53 (2.08–3.16) | 0.772 |
| HDL–C (mmol/L) | 1.16 (0.98–1.39) | 1.18 (0.99–1.42) | 1.20 (0.99–1.44) | 1.19 (0.98–1.47) | 0.233 |
| Apolipoprotein E (mg/L) | 36.8 (31.7–43.5) | 36.6 (31.5–43.1) | 36.8 (31.8–43.0) | 35.9 (30.5–42.2) | 0.746 |

SBP, systolic blood pressure; DBP, diastolic blood pressure; FBG, fasting blood glucose; ALT, alanine aminotransferase; AST, aspartate aminotransferase; ALP, alkaline phosphatase; γ-GTT, γ-glutamyltransferase; LDL-C, low-density lipoprotein cholesterol; HDL-C, high-density lipoprotein cholesterol.

^a^ Data are median (interquartile range) or N (%) as indicated.

^b^ FIB-4 index is a simple noninvasive index to predict liver fibrosis and is calculated as age (years)×AST [U/L]/(platelets [10^9^/L] × (ALT [U/L])^1/2^).
