# Supplementary material for: ABO blood group and risk of newly diagnosed nonalcoholic fatty liver disease: A case-control study in Han Chinese population
Source: PLoS One. 2019 Dec 4;14(12):e0225792. doi: 10.1371/journal.pone.0225792 (PMC6892526; doi:10.1371/journal.pone.0225792)
Supplement: S3 Table — (DOC) [file pone.0225792.s004.doc]

| **S3 Table.** Results of univariate and multivariate analyses on ABO blood group and NAFLD risk in patients with complete data | | | | | | | |
| --- | --- | --- | --- | --- | --- | --- | --- |
| Blood group | Cases | Controls | Odds ratio (95% confidence interval) | | | | |
|  |  |  | Unadjusted | Model 1 | Model 2 | Model 3 | Model 4 |
| O | 153 | 634 | 1.00 (reference) | 1.00 (reference) | 1.00 (reference) | 1.00 (reference) | 1.00 (reference) |
| Non-O | 339 | 1123 | 1.25 (1.01–1.55) | 1.29 (1.04–1.61) | 1.63 (1.26–2.11) | 1.59 (1.23, 2.05) | 1.60 (1.23, 2.07) |
| A | 167 | 548 | 1.26 (0.99–1.62) | 1.31 (1.02–1.69) | 1.58 (1.18–2.13) | 1.57 (1.16, 2.14) | 1.56 (1.14, 2.12) |
| B | 141 | 437 | 1.34 (1.03–1.73) | 1.37 (1.05–1.78) | 1.81 (1.32–2.47) | 1.80 (1.31, 2.48) | 1.80 (1.30, 2.48) |
| AB | 31 | 138 | 0.93 (0.61–1.43) | 0.98 (0.63–1.50) | 1.24 (0.74–2.09) | 1.26 (0.75, 2.13) | 1.29 (0.76, 2.17) |

Model 1 adjusted for age (continuous) and sex (male, female). Model 2 further adjusted for education level (low, medium, high), BMI (continuous), diabetes (yes, no), hypertension (yes, no), smoking status (current, past, never), and triglycerides (continuous). Model 3 adjusted for all variables selected by the change-in-estimate criterion [for non-O blood groups and NAFLD risk: BMI (continuous), diabetes (yes, no), smoking status (yes, no), ALT (continuous), γ-GTT (continuous), triglycerides (continuous), HDL-C (continuous), and apolipoprotein E (continuous); for blood group A, B and AB and NAFLD risk: education level (low, medium, high), BMI (continuous), diabetes (yes, no), hypertension (yes, no), smoking status (current, past, never), FBG (continuous), albumin (continuous), ALT (continuous), AST (continuous), ALP (continuous), γ-GTT (continuous), triglycerides (continuous), HDL-C (continuous), LDL-C (continuous), and apolipoprotein E (continuous)]. Model 4 adjusted for all variables mentioned above as well as FIB-4 index (continuous).
