# Supplementary material for: ABO blood group and risk of newly diagnosed nonalcoholic fatty liver disease: A case-control study in Han Chinese population
Source: PLoS One. 2019 Dec 4;14(12):e0225792. doi: 10.1371/journal.pone.0225792 (PMC6892526; doi:10.1371/journal.pone.0225792)
Supplement: S4 Table — The results of subgroup analyses on the associations of blood groups A, B and AB with the risk of NAFLD. (DOC) [file pone.0225792.s005.doc]

| **S4 Table.** The results of subgroup analyses on the associations of blood groups A, B and AB with the risk of NAFLD | | | | | | |
| --- | --- | --- | --- | --- | --- | --- |
| Association | Blood group A and NAFLD risk | | Blood group B and NAFLD risk | | Blood group AB and NAFLD risk | |
| Subgroup | OR (95% CI) ^a^ | *P*_interaction_ | OR (95% CI) ^a^ | *P*_interaction_ | OR (95% CI) ^a^ | *P*_interaction_ |
| Overall | 1.50 (1.13, 1.99) | - | 1.59 (1.19, 2.14) | - | 1.37 (0.86, 2.18) | - |
| Age (years) | | | | | | |
| ≥60 | 1.62 (1.06, 2.46) | 0.82 | 1.68 (1.09, 2.58) | 0.74 | 1.21 (0.63, 2.33) | 0.62 |
| <60 | 1.44 (0.97, 2.14) |  | 1.60 (1.06, 2.43) |  | 1.42 (0.72, 2.83) |  |
| Sex | | | | | | |
| Male | 1.48 (0.94, 2.32) | 0.52 | 1.72 (1.08, 2.72) | 0.78 | 1.20 (0.57, 2.50) | 0.68 |
| Female | 1.67 (1.14, 2.44) |  | 1.53 (1.03, 2.27) |  | 1.40 (0.75, 2.62) |  |
| BMI (kg/m^2^) | | | | | | |
| ≥25 | 1.55 (1.07, 2.26) | 0.31 | 1.67 (1.13, 2.48) | 0.68 | 1.43 (0.74, 2.74) | 0.91 |
| <25 | 1.25 (0.82, 1.91) |  | 1.50 (0.98, 2.32) |  | 1.31 (0.69, 2.49) |  |
| Hypertension | | | | | | |
| Yes | 1.20 (0.78, 1.85) | 0.23 | 1.27 (0.80, 2.00) | 0.16 | 1.04 (0.51, 2.13) | 0.36 |
| No | 1.79 (1.22, 2.64) |  | 1.92 (1.29, 2.84) |  | 1.70 (0.91, 3.17) |  |
| Current smoking | | | | | | |
| Yes | 1.34 (0.60, 2.98) | 0.52 | 1.81 (0.83, 3.93) | 0.96 | 1.91 (0.57, 6.36) | 0.63 |
| No | 1.59 (1.17, 2.17) |  | 1.52 (1.09, 2.11) |  | 1.23 (0.74, 2.06) |  |
| Diabetes | | | | | | |
| Yes | 1.29 (0.73, 2.27) | 0.48 | 1.29 (0.72, 2.32) | 0.56 | 0.89 (0.37, 2.16) | 0.19 |
| No | 1.55 (1.10, 2.18) |  | 1.75 (1.24, 2.48) |  | 1.65 (0.96, 2.85) |  |
| ALT (U/L) | | | | | | |
| >40 | 1.00 (0.43, 2.31) | 0.47 | 1.37 (0.55, 3.38) | 0.78 | 0.68 (0.18, 2.61) | 0.35 |
| ≤40 | 1.65 (1.21, 2.25) |  | 1.72 (1.24, 2.37) |  | 1.66 (1.01, 2.72) |  |
| FBG (mmol/L) | | | | | | |
| ≥7.0 | 1.97 (1.00, 3.88) | 0.51 | 1.61 (0.84, 3.09) | 0.95 | 0.82 (0.24, 2.74) | 0.48 |
| <7.0 | 1.49 (1.08, 2.05) |  | 1.60 (1.14, 2.24) |  | 1.51 (0.91, 2.51) |  |
| Triglycerides (mmol/L) | | | | | | |
| ≥1.7 | 1.94 (1.27, 2.96) | 0.15 | 1.28 (0.82, 2.01) | 0.21 | 1.81 (0.90, 3.65) | 0.61 |
| <1.7 | 1.32 (0.88, 1.99) |  | 1.86 (1.25, 2.77) |  | 1.18 (0.62, 2.24) |  |

NAFLD, nonalcoholic fatty liver disease; OR, odds ratio; CI, confidence interval; BMI, body mass index; ALT, alanine aminotransferase; FBG, fasting blood glucose.

^a^ Adjusted for age (continuous), sex (male, female), educational level (low, medium, high), body mass index (continuous), diabetes (yes, no), hypertension (yes, no), coronary heart disease (yes, no), smoking status (current, past, never), prothrombin time (continuous), fasting blood glucose (continuous), albumin (continuous), AST (continuous), ALT (continuous), alanine aminotransferase (continuous), γ-GTT, bile acids (continuous), triglycerides (continuous), HDL-C (continuous), LDL-C (continuous), apolipoprotein E (continuous), and FIB-4 index (continuous). In each case, the model is not adjusted for the stratification factor.
