# Supplementary material for: ABO blood group and risk of newly diagnosed nonalcoholic fatty liver disease: A case-control study in Han Chinese population
Source: PLoS One. 2019 Dec 4;14(12):e0225792. doi: 10.1371/journal.pone.0225792 (PMC6892526; doi:10.1371/journal.pone.0225792)
Supplement: S5 Table — (DOC) [file pone.0225792.s006.doc]

| **S5 Table.** Demographic, clinical and laboratory characteristics of patients after multiple imputation and propensity score matching | | | |
| --- | --- | --- | --- |
| Characteristics ^a^ | Cases (N=468) | Controls (N=468) | *P* |
| Demographics | | | |
| Age (years) | 59 (48-65) | 60 (49-69) | 0.123 |
| Male | 195 (41.7) | 197 (42.1) | 0.895 |
| Education | | | |
| Low | 88 (18.8) | 112 (23.9) | 0.113 |
| Medium | 267 (57.1) | 240 (51.3) |
| High | 113 (24.1) | 116 (24.8) |
| Body mass index (kg/m^2^) | 25.4 (23.6-27.3) | 25.3 (23.6-27.2) | 0.876 |
| Clinical features | | | |
| SBP (mm Hg) | 127 (118-139) | 129 (119-140) | 0.321 |
| DBP (mm Hg) | 78 (70-84) | 79 (70-86) | 0.125 |
| Smoking status | | | |
| Never | 369 (78.8) | 369 (78.8) | 0.984 |
| Past | 18 (3.9) | 19 (4.1) |
| Current | 81 (17.3) | 80 (17.1) |
| Coronary heart disease | 113 (24.2) | 123 (26.3) | 0.452 |
| Hypertension | 210 (44.9) | 223 (47.6) | 0.394 |
| Diabetes | 131 (27.9) | 135 (28.9) | 0.772 |
| Laboratory tests | | | |
| Platelet count (10^9^/L) | 195 (158-232) | 195 (162-236) | 0.522 |
| FBG (mmol/L) | 5.57 (4.94-6.92) | 5.50 (4.88-6.74) | 0.872 |
| ALT (U/L) | 24 (17-36) | 20 (14-30) | 0.538 |
| AST (U/L) | 22 (18-27) | 21 (18-27) | 0.821 |
| FIB-4 index ^b^ | 1.34 (0.94-1.84) | 1.40 (0.99-1.98) | 0.575 |
| ALP (U/L) | 78 (66-92) | 76 (64-92) | 0.357 |
| γ-GTT (U/L) | 28 (21-45) | 25 (18-45) | 0.603 |
| Albumin (g/L) | 42.9 (40.7-45.0) | 42.8 (40.9-44.9) | 0.548 |
| Prothrombin time (s) | 12.9 (12.5-13.3) | 12.9 (12.4-13.4) | 0.582 |
| Total bilirubin (umol/L) | 10.5 (8.4-13.3) | 10.1 (8.1-12.8) | 0.578 |
| Bile acid (umol/L) | 3.6 (2.1-6.1) | 3.5 (2.1-5.9) | 0.914 |
| Triglycerides (mmol/L) | 1.70 (1.27-2.42) | 1.57 (1.15-2.33) | 0.977 |
| Total cholesterol (mmol/L) | 4.72 (4.14-5.43) | 4.78 (4.08-5.54) | 0.748 |
| LDL-C (mmol/L) | 2.77 (2.28-3.38) | 2.83 (2.21-3.43) | 0.820 |
| HDL-C (mmol/L) | 1.08 (0.92-1.27) | 1.08 (0.93-1.25) | 0.861 |
| Apolipoprotein E (mg/L) | 38.6 (33.3-45.6) | 38.4 (32.9-45.4) | 0.837 |

SBP, systolic blood pressure; DBP, diastolic blood pressure; FBG, fasting blood glucose; ALT, alanine aminotransferase; AST, aspartate aminotransferase; ALP, alkaline phosphatase; γ-GTT, γ-glutamyltransferase; LDL-C, low-density lipoprotein cholesterol; HDL-C, high-density lipoprotein cholesterol.

^a^ Data are median (interquartile range) or N (%) as indicated.

^b^ FIB-4 index is a simple noninvasive index to predict liver fibrosis and is calculated as age (years)×AST [U/L]/ (platelets [10^9^/L] × (ALT [U/L])^1/2^).
