# Supplementary material for: ABO blood group and risk of newly diagnosed nonalcoholic fatty liver disease: A case-control study in Han Chinese population
Source: PLoS One. 2019 Dec 4;14(12):e0225792. doi: 10.1371/journal.pone.0225792 (PMC6892526; doi:10.1371/journal.pone.0225792)
Supplement: S6 Table — (DOC) [file pone.0225792.s007.doc]

| **S6 Table.** Results of propensity score analyses on ABO blood group and risk of nonalcoholic fatty liver disease in patients with complete data | | | | | | |
| --- | --- | --- | --- | --- | --- | --- |
| Blood group | Propensity-score-adjusted ^a^ | | | Propensity-score-matched | | |
|  | Cases | Controls | OR (95% CI) | Cases | Controls | OR (95% CI) |
| O | 153 | 634 | 1.00 (reference) | 122 | 164 | 1.00 (reference) |
| Non-O | 339 | 1123 | 1.61 (1.24, 2.10) | 277 | 235 | 1.58 (1.18, 2.12) |
| A | 167 | 548 | 1.54 (1.14, 2.09) | 138 | 119 | 1.56 (1.11, 2.19) |
| B | 141 | 437 | 1.81 (1.31, 2.49) | 114 | 87 | 1.76 (1.22, 2.54) |
| AB | 31 | 138 | 1.29 (0.77, 2.17 | 25 | 29 | 1.16 (0.65, 2.08) |

OR, odds ratio; CI, confidence interval.

^a^ Propensity score was adjusted as a continuous variable in the regression model.
